# Endovascular treatment of a large saccular aneurysm of the celiac artery: a case report and review of literature

**DOI:** 10.1093/jscr/rjab437

**Published:** 2021-10-13

**Authors:** Javad Salimi, Amir Mangouri, Ali Farahanchi Baradaran

**Affiliations:** Department of Vascular and Endovascular Surgery, Sina Hospital, Tehran University of Medical Sciences, Tehran, Iran; Department of Vascular and Endovascular Surgery, Sina Hospital, Tehran University of Medical Sciences, Tehran, Iran; Department of Vascular and Endovascular Surgery, Sina Hospital, Tehran University of Medical Sciences, Tehran, Iran

## Abstract

Celiac artery is a visceral abdominal vasculature whose aneurysms are very rare, accounting for less than 0.01% of all aneurysms. This condition can be treated by open aneurysmectomy or aneurysmorrhaphy and endovascular intervention. Due to the high mortality and morbidity associated with open surgery, endovascular intervention may be a better treatment option. Here, we present a case related to a 40-year-old man who had been experiencing vague epigastric pain for 4 months prior to admission and was managed endovascularly.

## INTRODUCTION

The celiac artery is a visceral abdominal vasculature with very few aneurysms, accounting for less than 0.01% of all aneurysms. Many patients with celiac artery aneurysm are diagnosed after they die due to the aneurysm rupture [1, 2]. The aneurysm rupture rate can be reduced from 87 to 7% by improving diagnostic and intervention facilities [3]. Open aneurysmectomy or aneurysmorrhaphy, as well as endovascular intervention, can be used to treat this disease. Because of the high mortality and morbidity associated with open surgery, endovascular intervention may be a better treatment option [4].

## CASE REPORT

The patient, a 40-year-old man, had been complaining of vague epigastric pain for 4 months before admission. During the previous 4 months, he was treated with proton pump inhibitor drugs. His pain worsened 7 days before admission. Color Doppler Sonography revealed a celiac artery aneurysm. The patient’s medical history revealed blunt abdominal trauma 4 years ago that had gone unnoticed until 4 months before admission. He had no history of hypertension, diabetes mellitus or smoking. There was no family history of vascular or rheumatologic disease. He had no prior surgical or allergic history. His blood pressure was 120/80, his heart rate was 86 and he had only mild tenderness in the epigastric area with no rebound tenderness.

CT angiography revealed a saccular aneurysm in the right trunk of the celiac artery (42*56*59 mm) with thrombus formation lining its wall. The aneurysm lumen was patent and had a diameter of 20*21*34 mm. With a 12-mm patent neck, the aneurysm was connected to the main artery. The aneurysm was surrounded by distal branches of the left gastric, common hepatic and splenic arteries. A percutaneous angiographic intervention was suggested to the patient, and he was asked to go to NPO from midnight. After prepping and draping, a right femoral artery puncture was performed under local anesthesia, and a 6 French angiographic sheath (Arrow®) was inserted. The 6 French JR catheter (Alvision™) was extended to the aorta via a hydrophilic wire (AqWire®) and contrast media was injected, revealing a celiac artery aneurysm. The 8F*40 (Arrow®) sheath was then replaced, and the celiac artery was cannulated, and two 40*10 and 40*9 mm covered stents (Fluency™) were inserted through a stiff park wire (Amplatz Emerald). Finally, good results were obtained, with full aneurysmal coverage and distal celiac artery patency. Three days after the patient was admitted to the ward, a CT angiography revealed good results with complete coverage of the aneurysmal area and no leakage.

**Figure 1:**
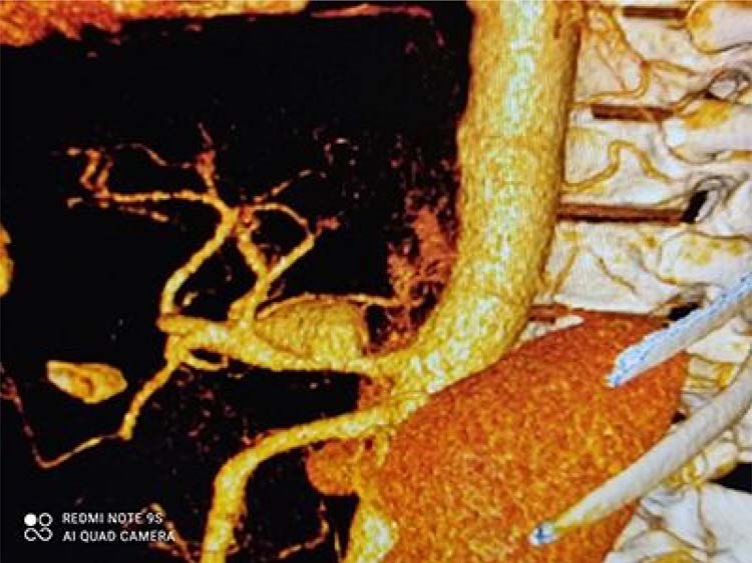
3D dimensional CT angiography shows CAA before intervention.

**Figure 2:**
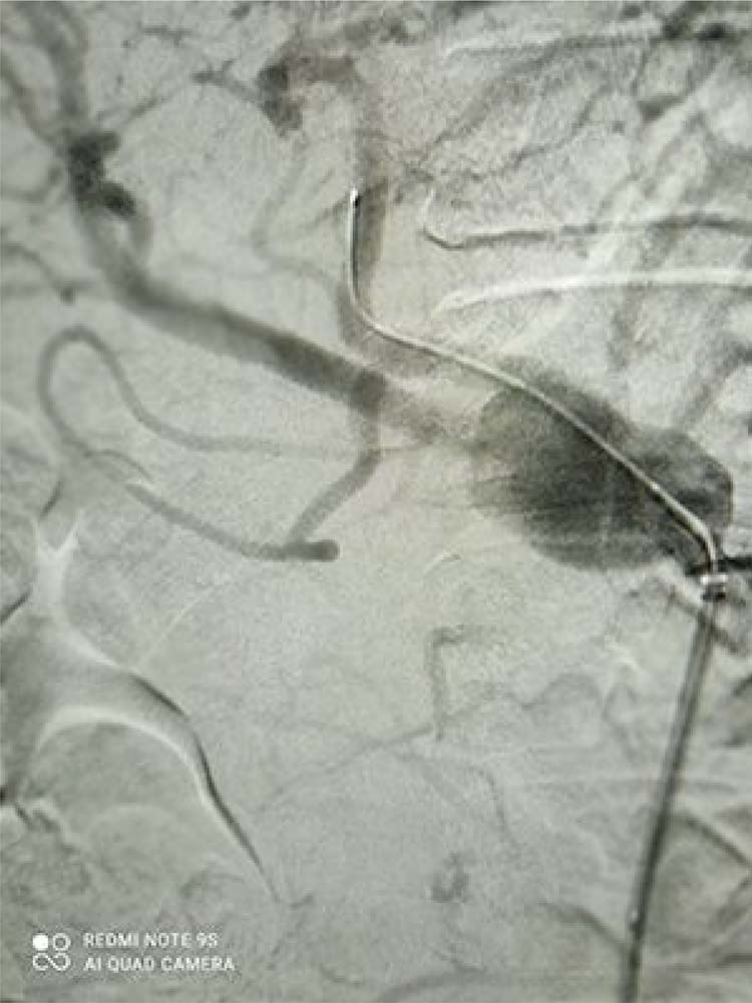
Angiography revealed large CAA.

Figures 1 and 2 depict CAA in 3D images of CT angiography and angiography, respectively.

**Figure 3:**
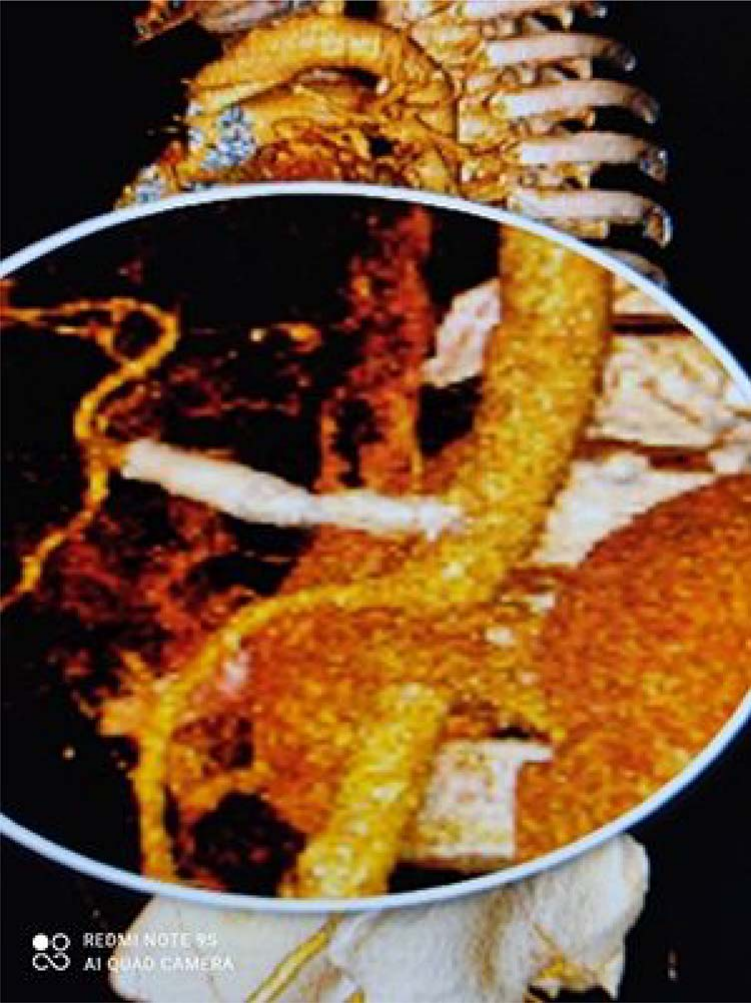
3D dimensional CT angiography shows CAA after intervention.

**Figure 4:**
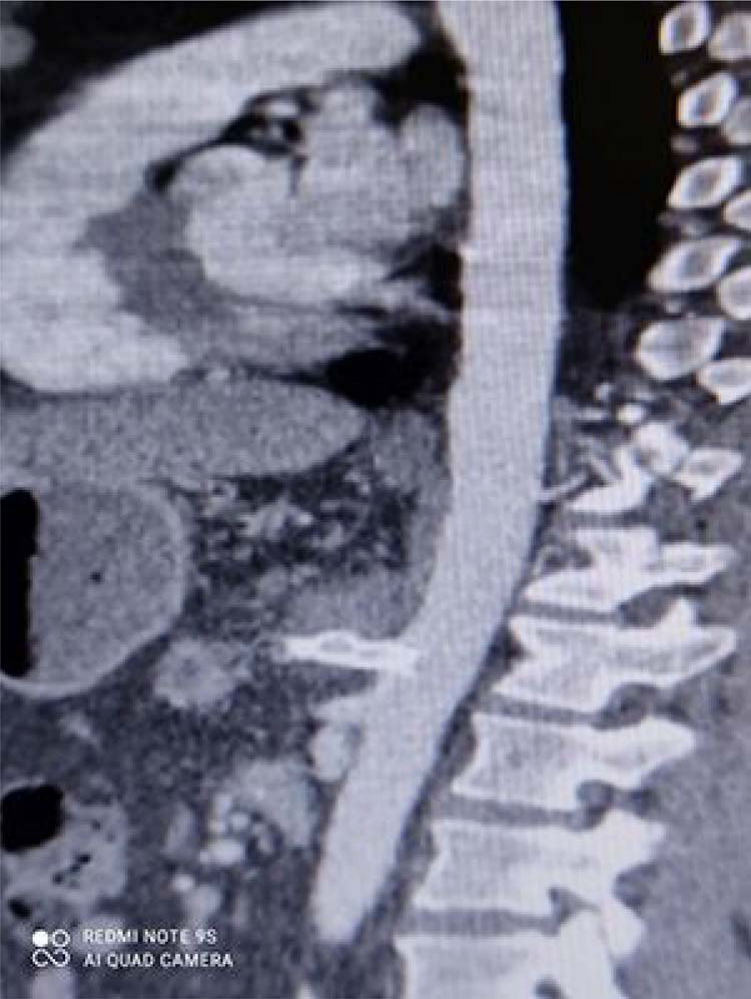
CT angiography showing repaired CAA with no leakage.

Figures 3 and 4 show CT angiography findings after the endovascular intervention.

## DISCUSSION

Here, we presented a case related to a 40-year-old man with vague epigastric pain who was finally diagnosed with a celiac artery aneurysm. The main risk factor for aneurysmal rupture appears to be a silent expansion of vague pain, which is not investigated prior to aneurysmal rupture causing severe pain [3]. When the celiac artery aneurysm is diagnosed, the type of early intervention depends on its size and whether or not it is painful. Some authors believe that an aneurysm that is rapidly growing or that is larger than three times the normal reference vessel diameter should be treated to avoid complications [5]. Endovascular and surgical approaches are two treatment options; endovascular intervention may be a better option for old patients who have comorbidities or are hemodynamically unstable. Surgery, on the other hand, may be a better option when there is significant tortuosity or evidence of an infected sac [6]. Atherosclerosis is the most common predisposing factor (30%), followed by trauma (21%) and inflammation (11%) due to infection, pancreatitis or previous surgery. Since atherosclerosis is the leading cause of aortic aneurysm, the coexistence of multiple aneurysms should be investigated [7, 8]. Because the patient described in this case report lacked any risk factors for atherosclerosis, abdominal blunt trauma 4 years before admission could have been the cause of his disease. Although gastrointestinal symptoms such as vague epigastric or abdominal pain, nausea and vomiting are common, they can lead to misdiagnosis [7]. When a patient exhibits vague abdominal pain and the previously mentioned risk factors, it is reasonable to evaluate the patient for splanchnic artery aneurysms. There are two types of disease: dissecting disease and saccular disease. The saccular form can be seen as an aneurysm with full wall thickness and a pseudoaneurysm with the destructed artery wall. According to Shukla *et al*., endovascular treatment for celiac artery aneurysm is associated with lower short-term complications (7.4% complications compared with 28.6% complications in surgery cases; *P* = 0.025) and higher long-term survival [9].

Tulsyan *et al*. [10] used coil and glue embolization in 48 procedures in patients with visceral artery aneurysms, including three celiac axis repairs, with a 98% success rate. The mean size of celiac axis branches in this study was 22 mm. Endovascular embolization was not recommended by Xia *et al*. [11] for patients with celiac artery aneurysms larger than 30 mm, and patients were treated with endovascular stent repair with a 100% success rate. Since the patient in this study had a relatively large aneurysm, we used stenting repair instead of embolization, but some reports disagree with this theory.

Borzelli *et al*. described a large aneurysm of the celiac trunk that was filled with thrombus and had a true lumen size of 22*27 mm. Transcatheter coil embolization and packing of the aneurysmal sac were performed successfully for the patient, but 1 month later, the patient complained of recurrent abdominal pain, and a second embolization was performed successfully with no complications [12].

Dwivedi *et al*. reported a 17-year-old boy with a history of Behcet’s disease who had a giant celiac aneurysm with a wide neck measuring 60 mm in diameter. Endovascular treatment was successful, with an aortic stent graft and transcatheter coil embolization of the aneurysm sac. They followed the patient for a year, and the control CT angiography revealed a complete aneurysm thrombus [13].

Vitale *et al*. [14] also used coil embolization to treat a celiac artery aneurysm in a 64-year-old man with coronary artery disease who presented with abdominal pain and a pulsatile mass.

Atkins *et al*. [4] discovered a 10-cm celiac artery aneurysm in a 61-year-old woman and inserted a stent graft within the abdominal aorta at the celiac orifice to exclude blood flow from the celiac artery aneurysm.

Al-Wahbi *et al*. [15] reported a 42-year-old man with an asymptomatic giant celiac artery aneurysm that received good collateral supply from the superior mesenteric artery. They followed the patient for 48 months after a transcatheter coil embolization, which revealed a completely thrombosed aneurysm with no symptoms in the patient.

Finally, it can be stated that stent grafting can be used to treat celiac artery aneurysms safely and effectively (Table 1).

**Table 1:** Presentation and management of recent cases

Author	Sex	Age	SXS	Aneurysm	Operation
Xia^*^	Male	87	Asymptomatic	10.5-cm CAA	Micro-Nester embolization coils used in the common hepatic artery
Atkins^*^	Female	61	Abdominal pain	10-cm CAA	Stent graft component within the abdominal aorta at the celiac artery orifice
Carrafiello^*^	Male	60	Asymptomatic	34^*^14 mm CAA	Two stents were inserted in the celiac trunk and in the splenic artery
Xia^*^	7 Male 4 Female		7 Patients with abdominal pain and 2 asymptomatic		Stenting of celiac and common hepatic arteries or just celiac artery
Borzelli^*^	Male	57	Severe abdominal pain	60 × 57 mm celiac trunk	Coils embolization
Dwivedi^*^	Male	17	Abdominal pain, nausea and vomiting (patient had Bechet’s disease)	60-mm CAA	Stent graft of the aorta and transcatheter coil embolization of the aneurysm sac
Vitale^*^	Male	64	Abdominal pain	13-cm CAA	Coil embolization of the splenic artery, occlusion of the origin of the celiac artery with an Amplatzer occluder device
Al-Wahbi^*^	Male	42	Asymptomatic	7.1 cm × 4.3 cm CAA	Coil embolization and packing of the aneurysm sack

^*^References: [2, 4, 6, 11–15].
